# Effect of scapular stabilization exercises on subacromial pain (impingement) syndrome: a systematic review and meta-analysis of randomized controlled trials

**DOI:** 10.3389/fneur.2024.1357763

**Published:** 2024-03-01

**Authors:** Ziyi Zhong, Wanli Zang, Ziyue Tang, Qiaodan Pan, Zhen Yang, Bin Chen

**Affiliations:** ^1^Institute of Life Course and Medical Sciences, University of Liverpool, Liverpool, United Kingdom; ^2^Postgraduate School, Harbin Sport University, Harbin, China; ^3^Department of Rehabilitation Medicine, The First Affiliated Hospital, Sun Yat-sen University, Guangzhou, China; ^4^School of Medicine, Tongji University, Shanghai, China; ^5^Department of Movement Sciences, KU Leuven-University of Leuven, Leuven, Belgium; ^6^Shanghai Yangzhi Rehabilitation Hospital (Shanghai Sunshine Rehabilitation Center), School of Medicine, Tongji University, Shanghai, China

**Keywords:** subacromial pain syndrome, scapula, scapular stabilization exercises, exercise therapy, systematic review, meta-analysis

## Abstract

**Objective:**

To evaluate the effectiveness of scapular stabilization exercises (SSE) in the treatment of subacromial pain syndrome (SAPS).

**Methods:**

Clinical randomized controlled trials (RCTs) on SSE in the treatment of SAPS were searched electronically in PubMed, Science Direct, Cochrane Central Register of Controlled Trials (CENTRAL), EBSCOhost, Physiotherapy Evidence Database (PEDro), Web of Science, and other databases from 2000 to 2022, supplemented by manual search. Final RCTs were selected based on inclusion and exclusion criteria, and the Physiotherapy Evidence Database scale was used to evaluate the methodological quality of the study. A meta-analysis was conducted on data using the RevMan5.4 software.

**Results:**

Eight RCTs involving 387 participants were included. The meta-analysis showed that the experimental group (SSE) had greater improvements in the Visual Analog Scale score [Weighted Mean Difference (WMD) = −0.94, 95% CI (−1.23, −0.65), *p* < 0.001] and the Shoulder Pain and Disability Index score [WMD = −10.10, 95% CI (−18.87, −1.33), *p* = 0.02] than the control group (conventional physical therapy). However, range of motion (ROM) was not found to be greater in the experimental group than in the control group.

**Conclusion:**

Existing evidence moderately supports the efficacy of SSE for reducing pain and improving function in SAPS, without significant improvement in ROM. Future research should focus on larger, high-quality, standardized protocols to better understand SSE’s effects across diverse SAPS populations, treatment, and outcome measures.

**Systematic Review Registration:**

https://www.crd.york.ac.uk/PROSPERO/display_record.php?RecordID=307437, CRD42022307437.

## 1 Introduction

Shoulder pain ranks as the second most prevalent musculoskeletal pain (1–3), with approximately 67% of adults experiencing shoulder pain (4). Subacromial pain syndrome (SAPS) is the most common shoulder disorder and significantly impacts physical functioning, mental health, and quality of life (5). The term “subacromial impingement syndrome” was coined by Neer to describe shoulder pain caused by the acromion exerting mechanical stress on the rotator cuff tendon during arm elevation (6). However, the terminology remains controversial (7). Diercks et al. (8) introduced the term “subacromial pain syndrome” as a more comprehensive and precise descriptor for chronic shoulder pain with diverse etiologies (9). SAPS is characterized by unilateral shoulder pain localized around the acromion, accompanied by limited range of motion (ROM) in abduction (10), adduction (10, 11), and internal rotation (IR) (11), in abduction (10).

Conservative management, particularly exercise therapy, is recommended as the initial approach for SAPS according to treatment guidelines (8, 12–14). Exercise therapy has shown effectiveness in relieving pain and improving SAPS-related dysfunction by targeting posture, muscle weakness, scapular stability, and scapulohumeral rhythms. However, the specific components of exercise programs for SAPS remain unclear due to program heterogeneity (15–21). Existing trials often suffer from limitations such as small sample sizes, short-term follow-up, and conflicting findings (15, 18, 22–25). Some studies have suggested that rehabilitation interventions for abnormal shoulder biomechanics should focus on the scapula (26–28). Scapular dyskinesia, characterized by altered shoulder kinematics, is frequently observed in patients with SAPS. These alterations may include increased scapular internal rotation (29) and anterior tilt (30, 31), as well as decreased upward rotation, retraction, and depression (29, 32). Scapular muscles play a crucial role in scapular positioning during rest and shoulder movements (33). In patients with SAPS, there is an underutilization of the middle and lower trapezius and serratus anterior muscles, while the upper trapezius muscle is overused (29, 34). Biomechanical factors, including tightness of the pectoralis minor, scapular retinaculum, and posterior capsule stiffness of the shoulder, are also associated with abnormal scapular position and may act as risk factors for SAPS (35). Considering these shoulder biomechanical abnormalities, scapula-centered rehabilitation interventions are now recommended (26–28). Scapular stabilization exercises (SSE) are a type of exercise therapy designed to restore scapular position and movement, enhance muscle function, and improve scapular kinematics. SSE, which emphasizes coordinated activation and co-activation of dynamic restraints, consist of various exercises such as wall slides with squats, wall push-ups with ipsilateral leg extension, lawnmower with diagonal squat, scapular-retraction exercises, and robbery with squat (36, 37). Although several studies have investigated SSE, the results have been inconsistent. While one systematic review (38) has explored this topic, research gaps remain, including the omission of grey literature and the limited number of included trials for quantitative synthesis. Moreover, new randomized controlled trials (RCTs) have been published since the initial literature search. Although adjunctive diagnostic tools such as dynamic ultrasound imaging show promise (39), the clinical tests commonly used for diagnosing SAPS have been found to have low accuracy and quality (40). Considering that physiotherapists prefer a pragmatic approach to managing SAPS based on the patient’s functional levels (41, 42), we selected shoulder pain and function as the primary outcome measures.

Accordingly, this comprehensive systematic review and meta-analysis aimed to determine the efficacy of SSE in improving pain and function in SAPS patients. We hypothesize that integrating SSE into clinical practice may enhance the prognosis of individuals with SAPS.

## 2 Methods

In the International Prospective Registry of Systematic Reviews (PROSPERO), the protocol of the present study was registered (ID: CRD42022307437). This review followed the Preferred Reporting Items of the Guide for Systematic Review and Meta-Analysis (PRISMA) (43).

### 2.1 Search strategy

The following electronic databases were searched: PubMed (MEDLINE), Science Direct, Cochrane Central Register of Controlled Trials (CENTRAL), EBSCOhost, the Physiotherapy Evidence Database (PEDro), and Web of Science. A combination of Medical Subject Headings terms (MeSH) and free text search terms was used for searching related articles. The unpublished research in the grey literature was extended through the Clinicaltrials.gov database, and the references of core articles were searched manually to identify other related articles. The search was limited to trials published in English. The retrieval period for all databases was from January 1, 2000, to May 1, 2022. The search strategy was initially formulated in PubMed and then adjusted and applied to other databases based on their respective characteristics. The detailed search strategy for all the databases is provided in Appendix 1. Zotero was used to create a bibliographic database to manage search results.

### 2.2 Selection criteria

The PICOS (population, intervention, comparison, outcome measure, study type) model was used to define the selection criteria:

#### 2.2.1 Types of population

Inclusion criteria consisted of (i) adults (age ≥18 years); and (ii) participants clinically diagnosed with SAPS or exhibiting typical characteristic symptoms, including a positive Neer test result or Hawkins–Kennedy test result (44, 45). Exclusion criteria included: (1) previous history of a shoulder injury, including acute trauma or shoulder operation, followed by post-operative treatment; (2) study focusing on other pathological changes in the shoulder joint complex except for SAPS, such as fracture/dislocation, glenohumeral joint instability, inflammatory arthritis, malignant tumors, etc.; (3) received a shoulder injection in the last month, shoulder or scapula focused exercise program.

#### 2.2.2 Types of interventions

(i) Treatment focused solely on SSE, or (ii) SSE in combination with other nonsurgical, nonpharmacological treatments or placebo treatments.

#### 2.2.3 Types of comparisons

Any nonsurgical, nonpharmacological treatments other than SSE (e.g., laser, ultrasound, extracorporeal shockwave therapy, or pulsed electromagnetic energy, corticosteroid injection, stretching, massage, manual therapy, physical factor therapy, exercise of the glenohumeral joint, glenohumeral joint mobilization, muscle strength training of the rotator cuff muscle and deltoid muscle, etc.), placebo treatment or blank control.

#### 2.2.4 Types of outcome measures

This systematic review focused on clinical efficacy outcomes related to SAPS, with the primary outcome being shoulder pain and function, and the secondary outcome being ROM.

#### 2.2.5 Types of studies

Randomized controlled trials and full-text articles published in English or with an attached English version were included.

### 2.3 Study selection process and data extraction

After literature retrieval, duplicate articles retrieved from different databases were excluded using Zotero. Two researchers independently reviewed the titles and abstracts, eliminating articles that did not meet the predefined inclusion criteria. A thorough analysis of the remaining articles was conducted to identify those eligible for inclusion in the systematic review. Disagreements between researchers were resolved through consultation and discussion. Nine articles were further examined to determine if they met the inclusion criteria and reached a consensus on inclusion. The result data for each selected study was extracted using a standardized table (Table 1) (55, 56). The collected information included author and publication year, country, participant demographics (number of participants, age, and sex), descriptions of the experimental and control groups, treatment frequency and duration, and outcome measurement.

**Table 1:** Main characteristics of the participants in each study.

Source	Country	Participants	Intervention	Comparison	Frequency & duration	Outcomes
Baskurt et al. (46)	Turkey	*n* = 40 IG: 20, CG: 20 13 m, 27 f Aged 24–71 yr Age (yr) mean: IG: 51.5 ± 8.4 CG: 51.3 ± 11.6	CG + SSE	Flexibility exercises (stretching) Strengthening exercises (rotator cuff & deltoid) Codman exercises Education & advice	6 weeks (3times/weeks)	-Pain: VAS -Shoulder ROM: flexion, abduction, IR (90°) and ER (90°) (an electronically goniometer) -Muscle strength (handheld dynamometer) -QoL: WORC -Joint Position Sense (JPS) -Scapular motion: LSST
Dabholkar et al. (47)	Belgium	*n* = 60 IG: 30, CG: 30 Age (yr) mean: IG: 54.34 ± 8.41 CG: 54.16 ± 7.61	CG + SSE	Conventional exercises Joint mobilizations	4 weeks (4 days/weeks)	-Function: QUICK DASH and PSFS -Pain: VAS
Hotta et al. (48)	Brazil	*n* = 60 IG: 30, CG: 30 18 m, 42 f Age (yr) mean: IG: 51 ± 8 CG: 47 ± 10	CG + SSE (retraction and depression)	Periscapular strengthening (upper trapezius, middle trapezius, lower trapezius, and serratus anterior)	8 weeks (3times/weeks)	-Function: SPADI -Pain: SPADI -Kinesiophobia -Global perceived effect -Satisfaction with treatment -Shoulder ROM (a digital inclinometer) -Scapula position -Muscle strength
Moezy et al. (49)	Iran	*n* = 68 IG: 33, CG: 35 13 m, 55 f Aged 21–78 yr Age (yr) mean: IG: 48.2 ± 13.8 CG: 47.8 ± 7.9	10 min walking warm-up on treadmill Stretching Strengthening exercises SSE Postural exercises	Physical modalities-infrared therapy, ultrasound therapy and transcutaneous Electrical nerve stimulation ROM exercises	6 weeks (3times/weeks)	-Pain: VAS -Shoulder ROM: ER and abduction (a standard goniometer) -Forward head posture -Mid-thoracic curve -Forward shoulder translation -Scapular protraction, rotation -Pectoralis minor length
Park et al. (50)	Korea	*n* = 30 IG: 15, CG: 15 7 m, 23 f Age (yr) mean: IG: 61.5 ± 7.7 CG: 61.0 ± 7.0	CG + SSE (scapula elevation, depression and retraction)	Heat treatment Ultrasound therapy Laser treatment Interferential current therapy	4 weeks (3times/weeks)	-Pain: VAS -Function: SST and CMS -Shoulder ROM (a goniometer)
Shah et al. (51)	India	*n* = 60 IG: 30, CG: 30 31 m, 29f Age (yr) mean: 46.93 IG: 46.9 CG: 46.96	CG + SSE	Strengthening exercises (shoulder flexors, abductors, horizontal abductors, external rotators) Stretching Wand exercises & pendulum exercises	4 weeks (6times/weeks)	-Pain: VAS -Pain and function: SPADI -Scapula movement: LSST
Struyf et al. (52)	Belgium	*n* = 22 IG: 10, CG: 12 10 m, 12 f Aged >18 yr Age (yr) mean: IG: 46.2 ± 13.5 CG: 45.4 ± 15.1	Stretching Scapular motor control training (trapezius and serratus anterior) Passive manual mobilization	Muscle friction Passive glenohumeral mobilizations Eccentric rotator cuff training Ultrasound therapy	9times (4–8 weeks) (1–3times/weeks)	-Function: SDQ -Diagnostic tests -Clinical tests: scapular positioning -Shoulder pain: VAS and VNRS -Muscle strength
Letafatkar et al. (53)	Iran	*n* = 80 IG: 37 (40), CG: 40 37 m, 43 f Age (yr) mean: IG: 40.5 ± 5.5 CG: 37.5 ± 6.3	CG+ Therapeutic exercise Three stretching and three strengthening exercises	No intervention Give a brochure about preventing overuse shoulder injuries and explaining how being active would relieve their symptoms	8 weeks (3times/weeks)	-Pain: VAS -Pain and function: DASH -Scapular kinematics: 3-dimensional motion software
Turgut et al. (54)	Turkey	*n* = 30 IG: 15 (18), CG: 15 (18) 16 m, 14 f Age (yr) mean: IG: 33.4 ± 9.3 CG: 39.5 ± 8.2	CG + SSE	Stretching (shoulder girdle) Strengthening exercises (rotator cuff strengthening)	12 weeks	-Scapular kinematics: 3-dimensional scapular kinematics -Pain: VAS -Function: SPADI

m, male; f, female; yr, years; IG, Intervention Group; CG, Control Group; SSE, scapular stabilization exercises; VAS, Visual Analogue Scale; QoL, Quality of Life; WORC, Western Ontario Rotator Cuff; LSST, the Lateral Scapular Slide Test; DASH, Disability of the Arm, Shoulder and Hand Questionnaire; PSFS, Patient-Specific Functional Scale; SPADI, Shoulder Pain and Disability Index; ROM, range of motion; ER, external rotation; IR, internal rotation; SST, Simple Shoulder Test; CMS, Constant-Murley Scale; SDQ, the Shoulder Disability Questionnaire; VNRS, Verbal Numeric Rating Scale.

### 2.4 Quality of assessment

The internal validity of each study was evaluated using the Physiotherapy Evidence Database (PEDro) scale, which includes 11 yes/no checklists to assess studies for allocation bias, blindness, and follow-up adequacy. Scores range from 0 to 10, with scores of 9–10 indicating excellent quality, 6–8 indicating good quality, 4–5 indicating fair quality, and scores below 4 indicating poor quality (57). The existing scores in the PEDro database were directly extracted, while the remaining trials were scored independently by two researchers. Any discrepancies in scores were resolved through discussion and consensus. Trials with scores below 4 were excluded. In addition, two researchers employed the Cochrane Handbook for Systematic Reviews of Interventions version 5.1.0 to assess the risk of bias in the included studies. The assessment criteria included: (1) selection bias; (2) performance bias; (3) detection bias; (4) reporting bias; and (5) other biases. The risk of bias was rated as “high,” “low,” or “unclear.” The quality of evidence was assessed using the Grading of Recommendations Assessment, Development, and Evaluation (GRADE) system, which evaluates five domains: study risk of bias, publication bias, indirectness, imprecision, and inconsistency. Grading level of “high,” “moderate,” “low,” or “very low” was assigned to each outcome.

### 2.5 Statistical analysis

Data analysis was performed using RevMan5.4 (Nordic Cochrane Centre, Copenhagen, Denmark). Sample sizes, post-intervention means, and standard deviations for the experimental and control groups were entered into the software. If means and standard deviations were missing, the authors of the articles were contacted for the necessary data. In this study, all outcome measures were continuous data, and the effect sizes were expressed with 95% confidence intervals (CIs), with a significance level of *p* < 0.05 indicating a statistically significant difference. When studies used different tools to measure a result, only the measurement results using the same tools would be selected to combine and analyze for the meta-analysis. The random-effects model was used to account for variability between studies and its impact on intervention. The heterogeneity of the included studies was analyzed, and *I*^2^ statistics were used to measure the heterogeneity between the included studies. Furthermore, the corresponding *p*-value was considered. When the *p*-value was ≥0.10 and *I*^2^ was ≤50%, the heterogeneity was considered to be small, and the fixed-effect model was used; when the *p*-value was less than 0.10 and *I*^2^ was >50%, the random-effect model was applied since studies differed greatly in terms of heterogeneity. A sensitivity analysis was used for studies with significant heterogeneity to identify the source or only descriptive analysis was adopted. If at least 10 studies were included in the meta-analysis, then the publication bias was estimated using a funnel chart.

## 3 Results

### 3.1 Study selection

**Figure 1:**
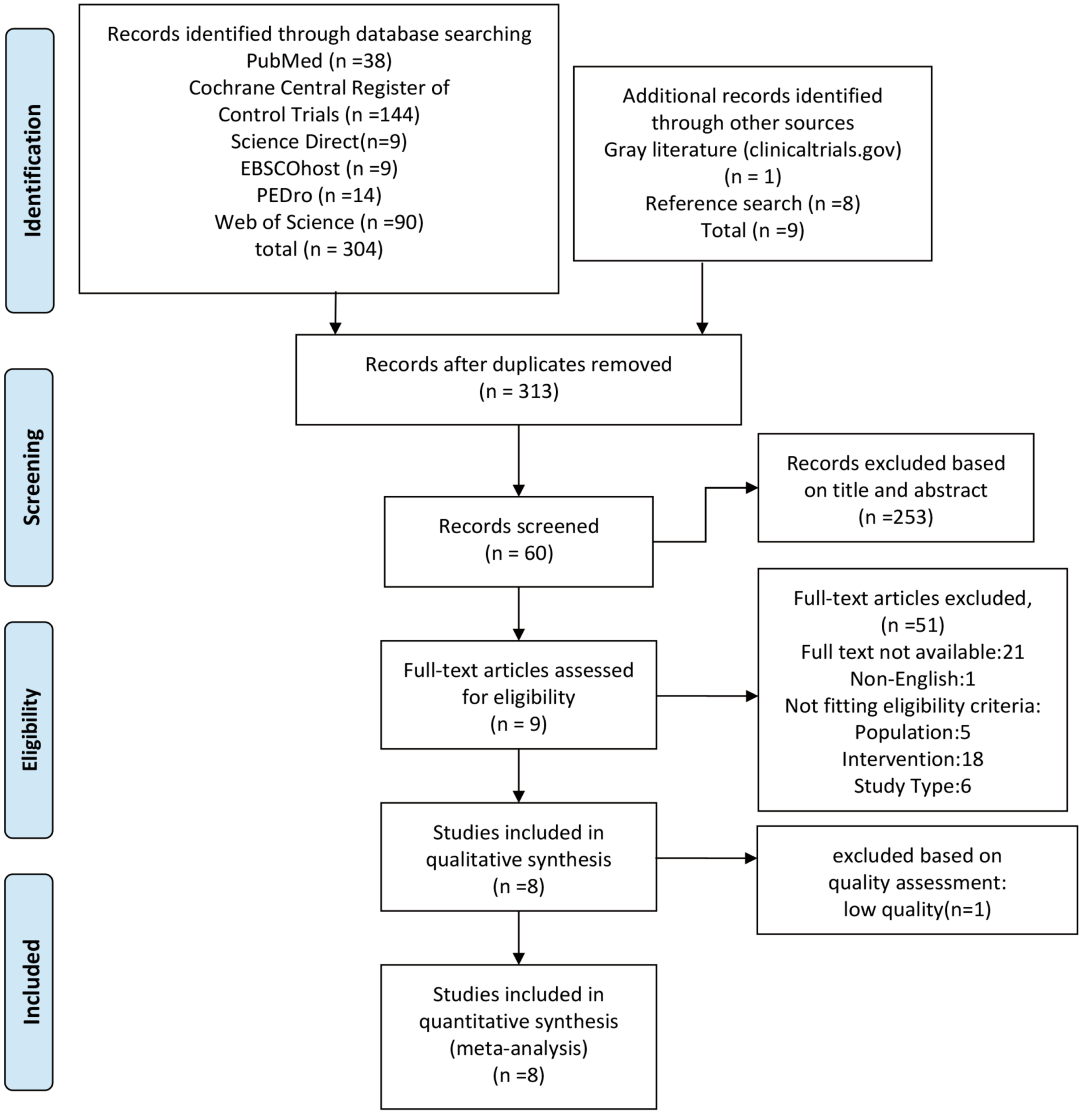
Flow chart: search and screening of the included studies.

The preliminary database search yielded 304 matches, and other approaches identified nine matches (including reference search), resulting in a total of 313 articles after removing duplicates (Figure 1). In screening the titles and abstracts, 253 studies were deemed irrelevant and excluded. Sixty studies underwent full-text screening, and nine studies met the inclusion criteria (46–51, 53, 54, 58, 59). One study (47) was excluded from the meta-analysis due to a low score (3 points), leaving eight studies for statistical comparison. A list of excluded studies during full-text screening, along with the reasons for exclusion, is provided in Appendix 2.

### 3.2 Study characteristics

Table 1 summarizes the basic characteristics of the nine included studies. A total of 387 participants were recruited, with sample sizes ranging from 22 to 77 participants in each study. Most articles reported the sex of the participants, except for one study (47) did not report the sex of 60 participants. The studies were conducted in various countries: Turkey (*n* = 2) (46, 54), Belgium (*n* = 2) (47, 52), Iran (*n* = 2) (49, 53), Brazil (*n* = 1) (48), and Korea (*n* = 1) (50). All articles were published between 2011 and 2021. The majority of studies had a training frequency of 3 times a week, while one study (51) had a frequency of 6 times a week. The training duration ranged from 4 weeks to 12 weeks.

### 3.3 Quality assessment

**Figure 2:**
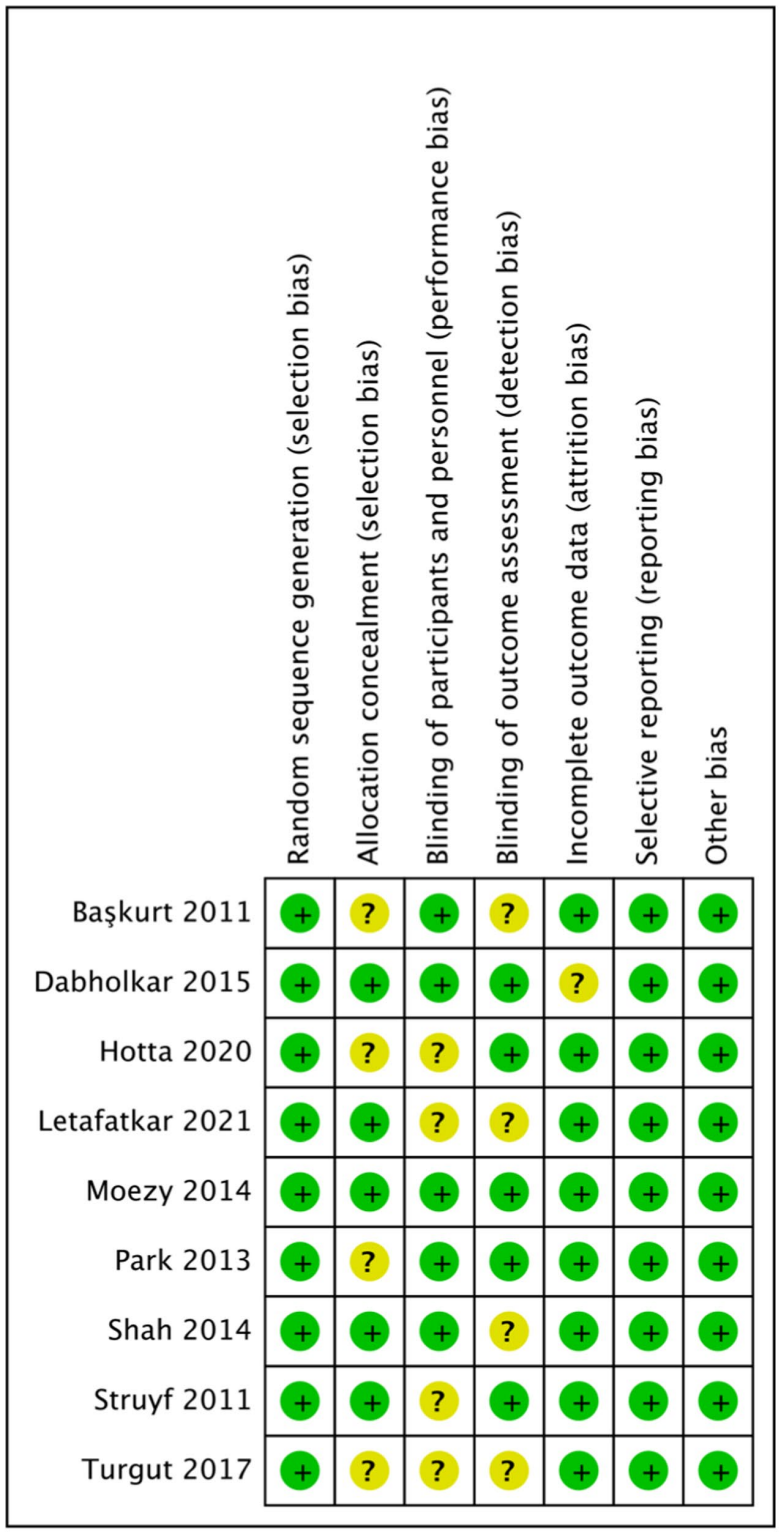
Flow chart: schematic representation of the methodological quality assessment of the literature in this study.

**Figure 3:**
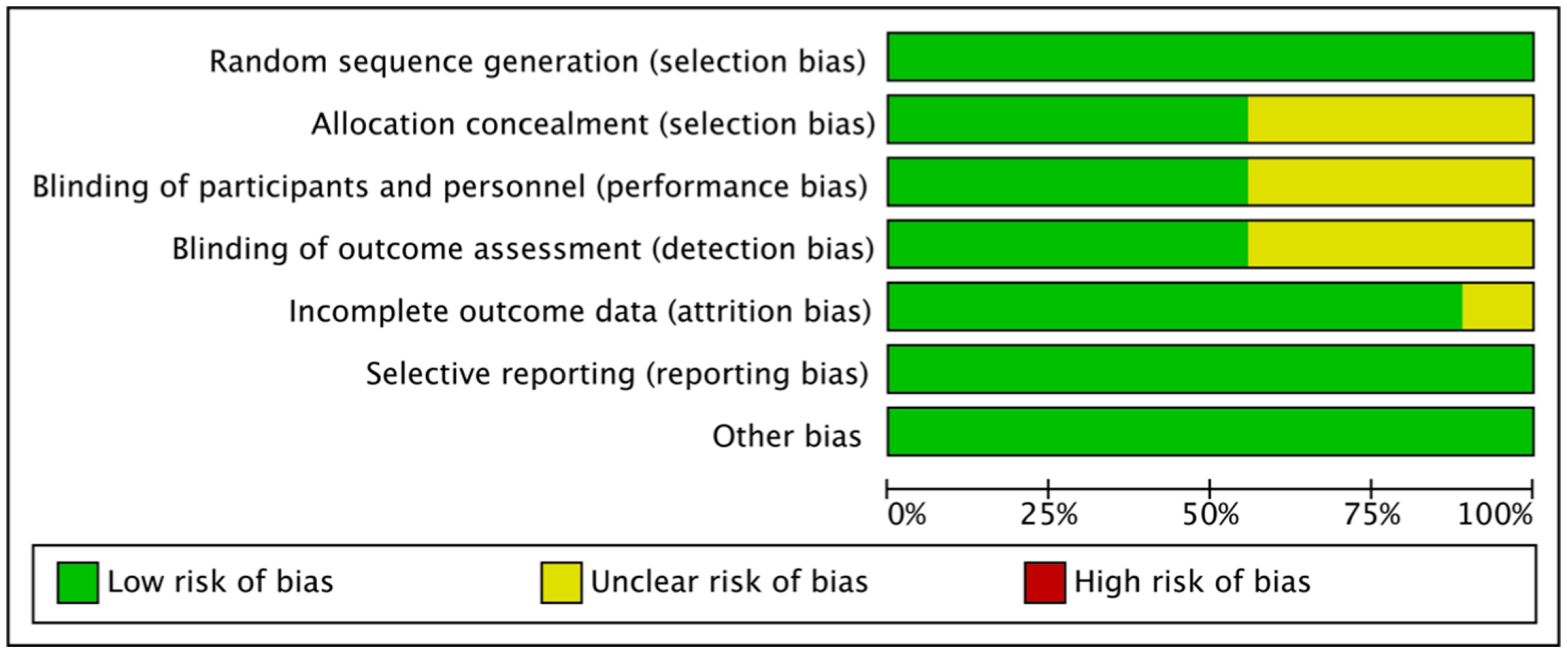
Flow chart: proportional representation of methodological quality assessment criteria in the literature of this study.

Table 2 presents the scores obtained using the PEDro scale. Among the studies with PEDro scale scores, four were rated as good, four as fair, and one study with a score below 4 was excluded from the calculation and subsequent meta-analysis (average PEDro total score = 5.875, range 4–8). The most common methodological flaws observed were inadequate concealment of distribution and therapist blindness, which may be attributed to the nature of the rehabilitation intervention (60). Additionally, the explanation for intention-to-treat analysis was unclear. There was a low risk of bias observed in random allocation, baseline comparability, between-group results, and point measures of variability in all of the studies; there was a low risk of bias for outcome data>85% in more than 75% of the studies. The risk of bias assessment for the included studies is illustrated in Figures 2, 3. Turgut et al. (54) presented the highest risk of bias, and Moezy et al. (49) presented the lowest risk of bias. The items obtained lower biases were the random sequence generation (selection bias), selective reporting (reporting bias) and other biases. The application of the GRADE system to assess the quality of evidence in the included studies revealed that the quality for each outcome ranged from “low” to “very low.” Detailed results are presented in Table 3.

**Table 2:** Methodological quality scores of included studies (The PEDro scale).

Study	1. Random allocation	2. Concealed allocation	3. Baseline comparability	4. Blinding subject	5. Blinding therapist	6. Blinding assessor	7. Outcome data >85%	8. Intention to treat	9. Between group results	10. Point measures/measures of variability	PEDro total score	Quality
Baskurt et al. (46)	1	0	1	0	0	0	1	0	1	1	5	Fair
Dabholkar et al. (47)	1	0	0	0	0	0	0	0	0	1	3	Low
Hotta et al. (48)	1	1	1	1	0	0	1	1	1	1	8	Good
Moezy et al. (49)	1	0	1	0	0	1	1	0	1	1	6	Good
Park et al. (50)	1	0	1	0	0	0	1	0	1	1	5	Fair
Shah et al. (51)	1	0	1	0	0	0	0	0	1	1	4	Fair
Struyf et al. (52)	1	0	1	0	0	1	1	1	1	1	7	Good
Letafatkar et al. (53)	1	1	1	0	0	1	1	1	1	1	8	Good
Turgut et al. (54)	1	0	1	0	0	0	0	0	1	1	4	Fair

PEDro, Physiotherapy Evidence Database. Item 1 is not used in the method score; 1 = Yes; 0 = No.

**Table 3:** The GRADE tool for the pooled results in the patients after concurrent training.

Outcomes		Illustrative comparative risks* (95% CI) Corresponding risk	Number of participants (studies)	Certainty of the evidence
Pain	VAS score	The VAS score of scapular stability training group was significantly better than that of control group [WMD = −0.81, 95% CI (−1.11, −0.51), *p* < 0.001]	327 (7 studies)	Low★+
Function	SPADI score	The SPADI score of scapular stability training group was significantly better than that of control group [WMD = −10.10, 95% CI (−18.87, −1.33), *p* = 0.02]	150 (3 studies)	Very low★‡+
ROM	Flexion	No significant difference in the active flexion range of the shoulder joint between groups [WMD = 1.20, 95% CI (−0.81, 3.21), *p* = 0.24]	65 (3 studies)	Low★‡
	Abduction	There was no significant difference in the flexion amplitude of shoulder joint movement between the two groups [WMD = 1.20, 95% CI (−0.81, 3.21), *p* = 0.24]	100 (4 studies)	Very low★‡+
	External rotation	After the treatment, there was no significant difference in ROM mobility between groups [WMD = 2.89, 95% CI (−3.30, 9.08), *p* = 0.36]	50 (4 studies)	Very low★‡+
	Internal rotation	The analysis of random-effect models revealed no significant differences between groups [WMD = −0.87, 95% CI (−6.04, 4.31), *p* = 0.74]	50 (2 studies)	Very low★‡+

### 3.4 Quantitative analysis

More than 10 measures were reported in the systematic review [Visual Analog Scale (VAS), ROM, muscle strength, Western Ontario Rotator Cuff, joint position test, muscle flexibility, neck and shoulder posture, Shoulder Pain and Disability Index (SPADI), disability of the arm, shoulder and hand questionnaire (DASH), three-dimensional scapular movement test, etc.] to evaluate the patients’ pain and level of disability, and inconsistencies among the obtained measurements hindered a summary of the results in the meta-analysis. VAS was used in seven studies to assess pain intensity. Several studies used different questionnaires to assess shoulder function, among which SPADI was the most commonly used. In addition, shoulder joint ROM was reported for both groups in several studies.

Seven of the eight studies were eligible for inclusion in the pain statistics set (46, 49–54), three were eligible according to the SPADI (48, 51, 54), and four were eligible according to the joint ROM (46, 48–50).

#### 3.4.1 Pain

**Figure 4:**
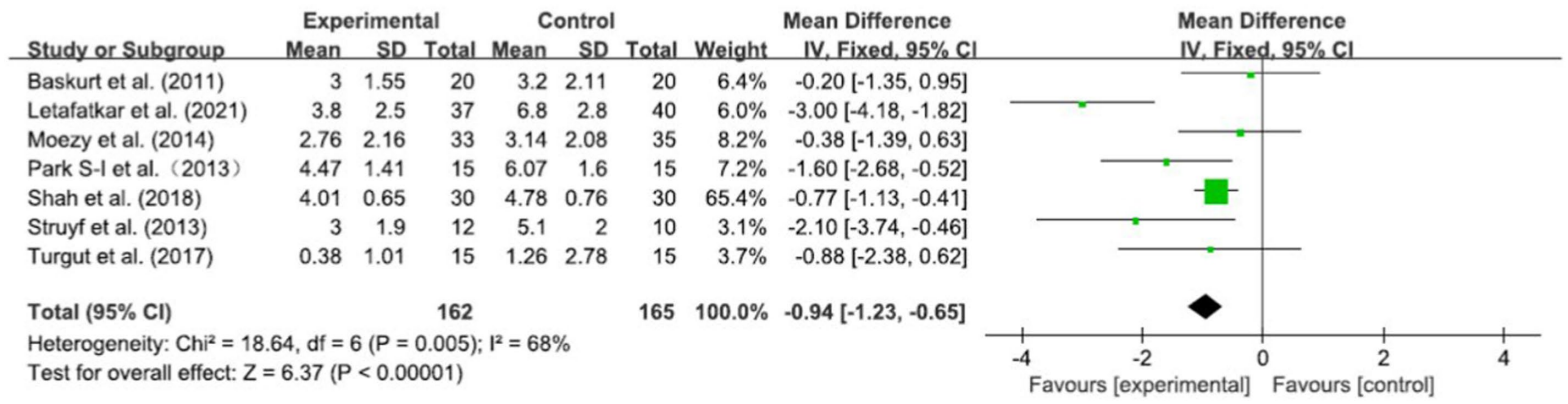
Forest plots: pain in the experimental group versus pain in the control group.

**Figure 5:**
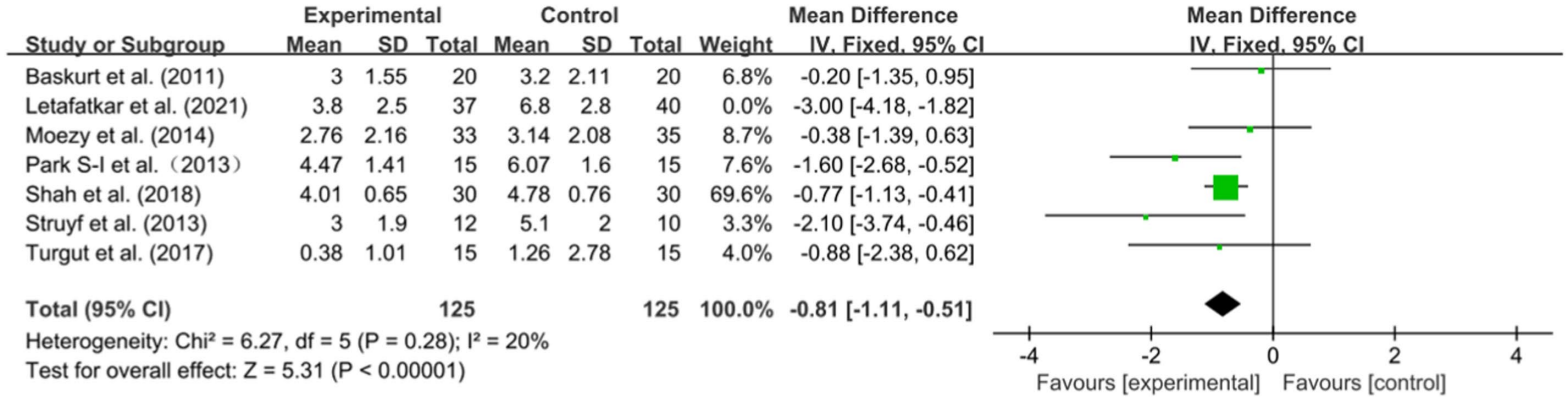
Forest plots: pain in the experimental group versus pain in the control group (sensitivity analysis).

The VAS scores of 327 patients were obtained in seven RCTs. After merging the data, the heterogeneity was obvious (*I*^2^ = 68%, *p* = 0.005). Based on a meta-analysis using a random-effect model, the VAS scores in the experimental group were significantly better than those in the control group [WMD = −0.81, 95% CI (−1.11, −0.51), *p* < 0.001] (Figure 4). To determine the reasons for the high heterogeneity, sensitivity analysis revealed a significant reduction in heterogeneity (*I*^2^ = 20%, *p* = 0.28) after deleting the study by Letafatkar et al. (53), while the deletion of any other research did not significantly affect the heterogeneity. Using fixed-effect model analysis, the results showed that the combined effect quantities were more stable [WMD = −0.94, 95% CI (−1.23, −0.65), *p* < 0.001] (Figure 5).

Hotta et al. (48) reported pain with the SPADI scale, while there was no significant difference between the experimental group and the control group, which was inconsistent with the results of the meta-analysis.

#### 3.4.2 Function

**Figure 6:**
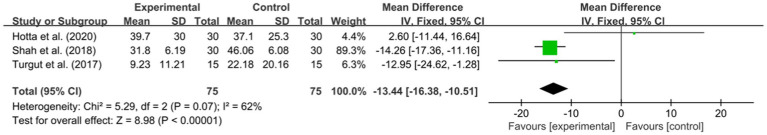
Forest plots: function in the experimental group versus function in the control group (SPADI).

**Figure 7:**
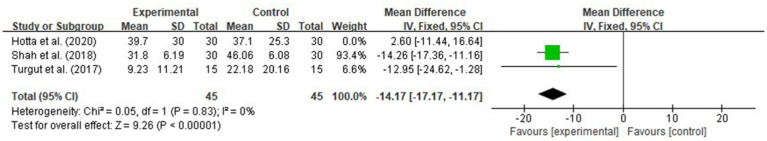
Forest plots: function in the experimental group versus function in the control group (SPADI) (sensitivity analysis).

SPADI scores were obtained in three RCTs involving a total of 150 participants. According to the heterogeneity test, there were obvious statistically significant differences between the studies (*I*^2^ = 62%, *p* = 0.07), so we chose the random-effect model. There was a significant difference in SPADI scores between the experimental and control groups based on a meta-analysis [WMD = −10.10, 95% CI (−18.87, −1.33), *p* = 0.02] (Figure 6). Similarly, according to the sensitivity analysis, heterogeneity was caused by including the Hotta et al. (48) study, however, heterogeneity decreased significantly after the study was deleted (*I*^2^ = 0%, *p* = 0.83). The fixed-effect model analysis showed that the results were more stable [WMD = −14.17, 95% CI (−17.17, −11.17), *p* < 0.001] (Figure 7).

Furthermore, functional measurements (non-SPADI) were performed in three RCTs, however, the measurement method used appeared only once and only a descriptive analysis was conducted. The research of Dabholkar et al. (47) showed that the Quick DASH and Patient Specific Functional Scale scores of the patients who performed SSE in the experimental group were better than those in the control group. Park et al. (50) showed that the Simple Shoulder Test and Constant-Murley Scale scores in the experimental group were better than those in the control group. The results of a study showed that the experimental group scored higher than the control group on the Shoulder Disability Questionnaire (34).

In summary, improvements in shoulder joint function differed significantly between groups.

#### 3.4.3 ROM

Meta-analysis showed that SSE had no obvious effect on improving shoulder joint ROM of patients with SAPS.

##### 3.4.3.1 Flexion

**Figure 8:**
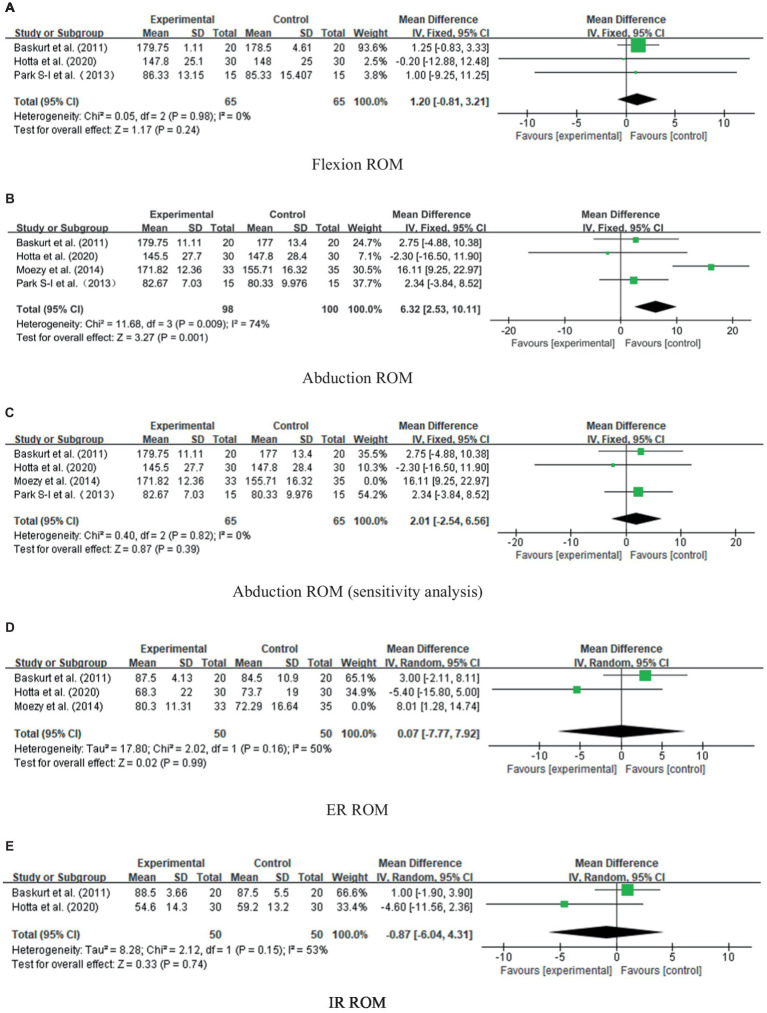
Forest plots: ROMs in **(A)** flexion, **(B)** abduction, **(C)** abduction ROM (sensitivity analysis), **(D)** ER and **(E)** IR in the experimental group versus those in the control group.

ROM measurement of shoulder flexion was performed in three RCTs (46, 48, 50) involving 130 participants. After the treatment, there was no significant difference in the active flexion range of the shoulder joint between groups [WMD = 1.20, 95% CI (−0.81, 3.21), *p* = 0.24], and no evidence of heterogeneity was found (*I*^2^ = 0%, *p* = 0.98). See Figure 8A.

##### 3.4.3.2 Abduction

In RCTs (46, 48–50) involving 198 participants, shoulder abduction ROM was measured. The meta-analysis indicated no significant difference between groups [WMD =5.52, 95% CI (−2.41, 13.45), *p* = 0.17] (Figure 8B), but the heterogeneity was high (*I*^2^ = 74%, *p* = 0.009). Sensitivity analysis showed that heterogeneity was caused by including the Moezy et al. (49) study, so heterogeneity decreased significantly after deletion (*I*^2^ = 0%, *p* = 0.82). The fixed-effect model analysis showed that the results were more stable [WMD = 2.01, 95% CI (−2.54, 6.56), *p* = 0.39], as presented in Figure 8C.

##### 3.4.3.3 External rotation

ROM measurement of external rotation (ER) of the shoulder joint was performed in 3 RCTs involving 168 participants (46, 48, 49). After the treatment, there was no significant difference in ROM mobility between groups [WMD = 2.89, 95% CI (−3.30, 9.08), *p* = 0.36], whereas there was significant heterogeneity between the studies (*I*^2^ = 56%, *p* = 0.10). Similarly, the heterogeneity was attributed to the study conducted by Moezy et al. (49). After deleting this study, the heterogeneity decreased (*I*^2^ = 50%, *p* = 0.16), and the result was more stable [WMD = 0.07, 95% CI (−7.77, 0.92), *p* = 0.99], as shown in Figure 8D.

##### 3.4.3.4 Internal rotation

ROM measurements of IR of the shoulder joint were performed in two RCTs (46, 48) involving 100 participants. The heterogeneity was high (*I*^2^ = 53%, *p* = 0.15), and the analysis of random-effect models revealed no significant differences between groups [WMD = −0.87, 95% CI (−6.04, 4.31), *p* = 0.74], as presented in Figure 8E. As a result of the limited number of documents included, only a descriptive analysis was performed. Both tests showed that the experimental and control groups were not significantly different.

### 3.5 Publication bias

For publication bias, according to the Cochrane recommendation, when the number of included studies was 10 or more, a funnel map was needed. A funnel chart analysis was not necessary because only eight studies were included in the systematic review and meta-analysis, thus publication bias could not be ruled out.

## 4 Discussion

This systematic review included eight randomized controlled trials and conducted a meta-analysis according to different outcome indicators, involving 387 participants, and evaluated the effectiveness of SSE in decreasing shoulder pain and reducing the level of disability in SAPS patients. Based on our research findings, SSE demonstrated clinical or statistical benefits when compared to conventional physical therapy, specifically in alleviating pain and enhancing functional outcomes. However, SSE did not show superior effectiveness in improving the ROM of the shoulder joint.

The meta-analysis of the VAS scores indicated a meaningful improvement in pain levels among participants in the experimental group, suggesting the clinical efficacy of the intervention in pain management. However, the observed heterogeneity in pain results across studies necessitated further scrutiny of the data. When one study (53) was excluded from the analysis, the heterogeneity was significantly reduced due to its substantial between-group difference. There are two likely causes for this: this study was unique as the control group did not receive any therapeutic intervention, and it had the longest training duration among all the studies. Furthermore, the meta-analysis of SPADI scores showed a notable improvement in the experimental group compared to the control group, indicating the effectiveness of the intervention as assessed by this scale. The observed reduction in shoulder pain following SSE is likely due to decreased stretching and tension in the cutaneous branches of the dorsal spinal nerve rami within the periscapular muscles, thereby mitigating myofascial pain of SAPS (61). It is also crucial to consider that sensory abnormalities and psychological factors may influence pain perception, and relying solely on the VAS may not provide a completely objective quantification of pain (62, 63). In addition, attentional focus instructions have been shown to enhance motor outcomes for patients with musculoskeletal disorders, guiding future intervention strategies (64).

When designing exercise tasks, it is recommended to provide patients with optimal attentional strategies to enhance their motivation and interest in the tasks (65). Moreover, physiotherapists should prioritize the psychological aspects and expectations of patients, enabling the implementation of a patient-centered treatment approach (66, 67).

Regarding the ROM of shoulder joint flexion, abduction, ER, and IR, no significant disparities were observed between the experimental and control groups. Notably, the exclusion of the study conducted by Moezy et al. (49) from the dataset resulted in a marked reduction in heterogeneity in the analysis of abduction and ER. This observation could potentially be explained by the specific ROM exercises included in the control group, which may have led to a more substantial enhancement in joint ROM for those participants. The reasons for the lack of significant improvement in shoulder joint ROM in our findings may be attributed to several factors. Firstly, the variation in measurement approaches for assessing ROM could potentially affect the comparability and consistency of the findings (68), with some (49, 50) employing manual goniometry and others utilizing devices. Moreover, recent literature suggests that SAPS is not a single diagnosis but rather a descriptive term encompassing various shoulder disorders with diverse symptoms (69, 70). This diversity in SAPS could significantly impact the outcomes reported in studies. SAPS is a multifactorial condition, encompassing anatomical-morphological aspects such as formation and abnormal growth of subacromial osteophytes and irregular acromion shape, as well as motor-biomechanical aspects of decreased rotator cuff muscle strength leading to upward displacement of the humeral head, and scapular movement dysfunction resulting from imbalances in the strength of scapular muscles (71–73). Another important confounding factor is the timing of patient inclusion and intervention, as delayed intervention is associated with joint capsule stiffness and slow recovery of active and passive ROM in the glenohumeral joint (74). The complexities arising from these factors highlight the necessity for continued research to elucidate critical aspects influencing the prognosis and progression of SAPS, as its natural history and influencing factors remain unclear (75, 76).

This study, while providing valuable insights, is not without its limitations. Firstly, the lack of universally accepted terminology or diagnostic criteria for SAPS patients leads to significant variations across studies and over time, resulting in heterogeneity within and between included patients (77). A recent scoping review (77) proposes identifying three subgroups of SAPS patients, which may help address this issue in future studies. Secondly, it is hypothesized that interventions may yield divergent effects at different stages of the condition. However, the absence of detailed reports on patient characteristics and SAPS classification precluded the possibility of conducting separate analyses for each stage. Furthermore, the limited sample size may restrict the generalizability of the findings.

## 5 Conclusion

In summary, the existing research provides moderate evidence supporting the efficacy of scapular stabilization exercises (SSE) in reducing pain and improving function for patients with subacromial pain syndrome (SAPS). However, it is important to note that these studies do not demonstrate a significant improvement in the range of motion (ROM). To gain a clearer understanding of SSE’s effects on different subgroups and stages of SAPS, future research should focus on conducting high-quality, large multicenter randomized controlled trials and standardizing protocols for SAPS population subgroups, treatment, and outcome measures.

## Data availability statement

The original contributions presented in the study are included in the article/Supplementary material, further inquiries can be directed to the corresponding authors.

## Author contributions

ZZ: Conceptualization, Data curation, Formal analysis, Investigation, Methodology, Project administration, Software, Writing – original draft. WZ: Conceptualization, Investigation, Project administration, Validation, Writing – original draft. ZT: Formal analysis, Validation, Writing – original draft. QP: Data curation, Formal analysis, Writing – original draft. ZY: Resources, Writing – review & editing. BC: Funding acquisition, Supervision, Writing – review & editing.
