# Supplementary material for: Effect of scapular stabilization exercises on subacromial pain (impingement) syndrome: a systematic review and meta-analysis of randomized controlled trials
Source: Front Neurol. 2024 Mar 1;15:1357763. doi: 10.3389/fneur.2024.1357763 (PMC10940535; doi:10.3389/fneur.2024.1357763)
Supplement: Supplementary file 1 [file Table_1.DOCX]

Supplementary Material

Effect of Scapular Stabilization Exercises on Subacromial Pain (Impingement) Syndrome: A Systematic Review and Meta-analysis of Randomized Controlled Trials

**Ziyi-Zhong1† Wanli Zang2† Zi-YueTang3 Qiaodan Pan4 Zhen Yang5* Bin Chen6***

**^*^Corresponding author:**

Bin-Chen

[chen_bin@tongji.edu.cn](mailto:chen_bin@tongji.edu.cn)

Zhen-Yang

zhen.yang@kuleuven.be

# Appendix 1. Detailed search strategies for all databases

(1) Search Strategy for Pubmed:

("subacromial impingement syndrome"[Title/Abstract] OR "impingement syndrome"[Title/Abstract] OR "subacromial impingement"[Title/Abstract] OR "impingement"[Title/Abstract] OR "shoulder impingement"[Title/Abstract] OR "rotator cuff tendinopathy"[Title/Abstract] OR "subacromial bursitis"[Title/Abstract] OR "rotator cuff tendonitis"[Title/Abstract] OR "rotator cuff tendinosis"[Title/Abstract] OR "supraspinatus tendonitis"[Title/Abstract] OR "shoulder dysfunction"[Title/Abstract] OR "contractile dysfunction"[Title/Abstract] OR "Shoulder Impingement Syndrome"[MeSH Terms]) AND ("scapula*"[Title/Abstract] AND ("stabilization"[Title/Abstract] OR "exercise*"[Title/Abstract] OR "rehabilitation"[Title/Abstract] OR "training"[Title/Abstract] OR "intervention"[Title/Abstract] OR "motor control"[Title/Abstract] OR "physiotherapy"[Title/Abstract] OR "physical therapy"[Title/Abstract] OR "Physical Therapy Modalities"[MeSH Terms] OR "Exercise Therapy"[MeSH Terms])) AND "randomized controlled trial"[Publication Type] AND 1990/01/01:2021/05/01[Date - Publication] [Filter]: randomized controlled trial [PT].

(2) Search Strategy for Cochrane:


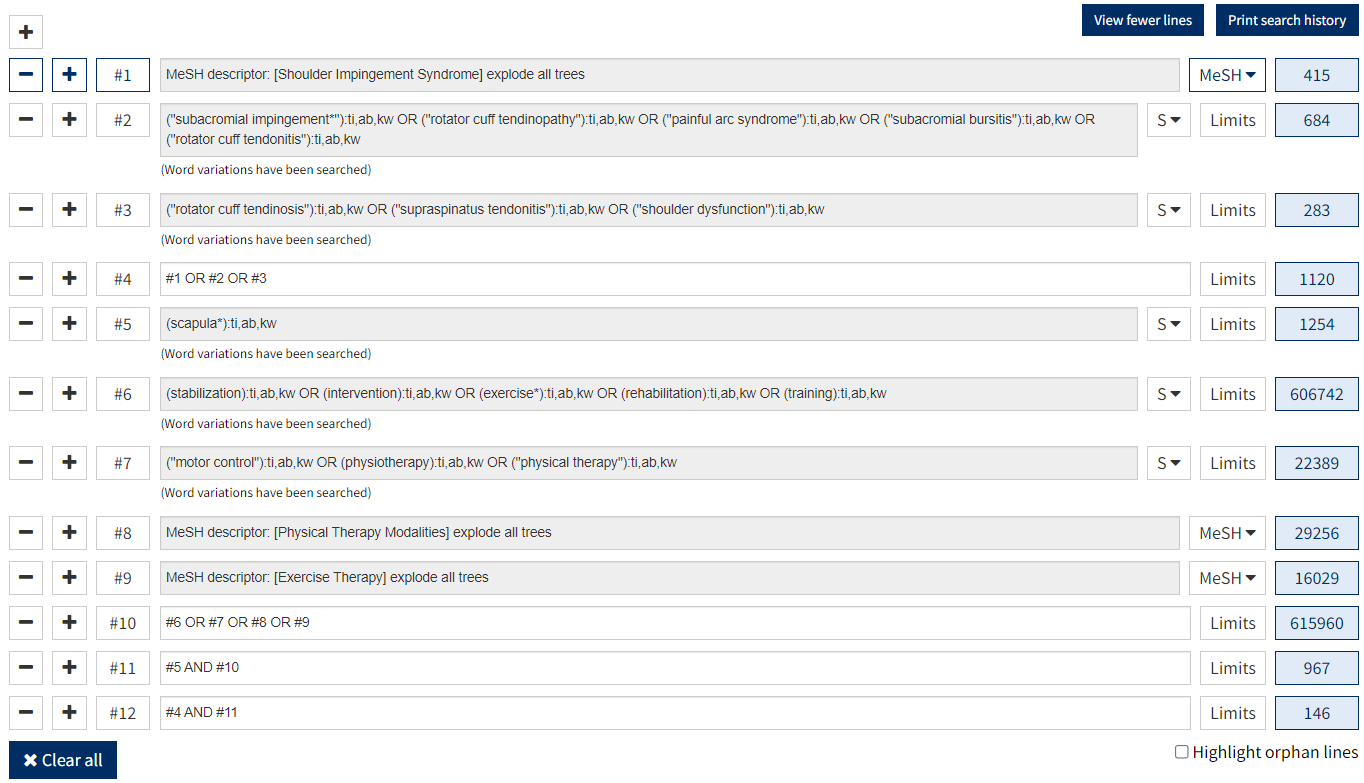


(3) Search Strategy for Science Direct:

(shoulder impingement syndrome OR sis OR shoulder tendinopathy OR rotator cuff tendinopathy OR subacromial impingement syndrome OR painful arc syndrome OR subacrominal bursitis) AND (scapular stabilization OR scapular strengthening)

Years: 2000-2022

Article Type: Research articles

(4) EBSCOhost

Business Source Premier ＆ SPORTDiscuss:

(shoulder impingement syndrome or sis or shoulder tendinopathy or rotator cuff tendinopathy or subacromial impingement syndrome) AND (scapular stabilization exercises or scapular strengthening exercise)

(5) PEDro


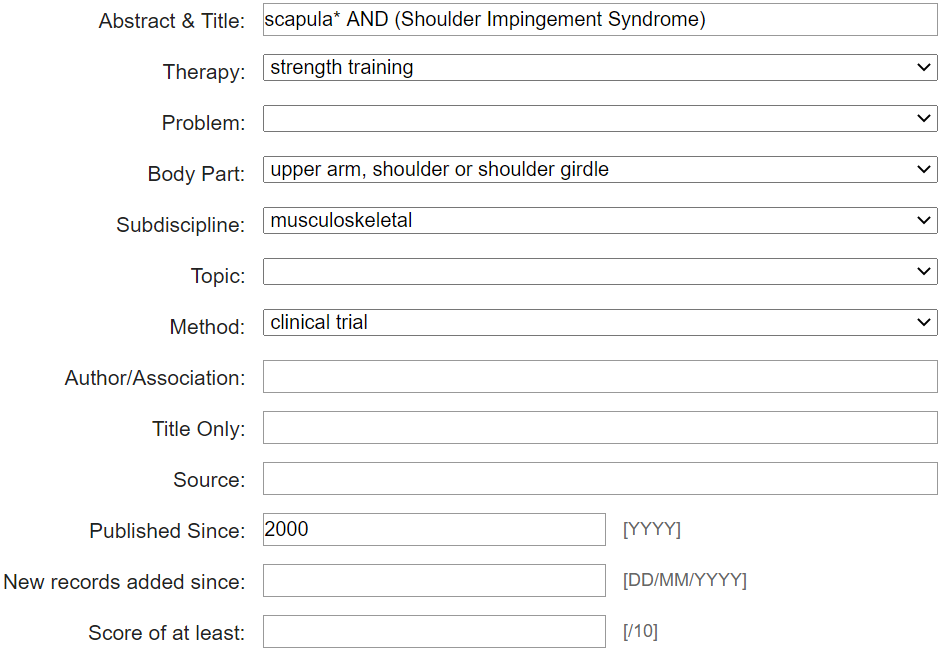


(6) Web of Science


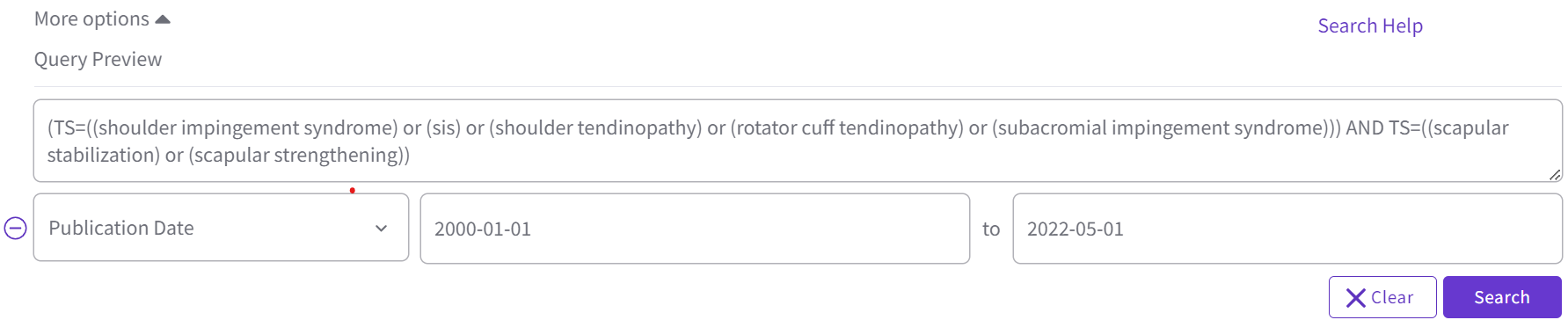


(TS=((shoulder impingement syndrome) or (sis) or (shoulder tendinopathy) or (rotator cuff tendinopathy) or (subacromial impingement syndrome))) AND TS=((scapular stabilization) or (scapular strengthening))

Publication Date：2000-01-01 to 2022-05-01.

# Appendix 2. Full-text articles excluded with motivations.

| Full text not available | 1. CTRI/2018/07/014843. Does kinetic control training have an effect on Pain and abnormal movement pattern around shoulder in Patients with Shoulder strains?*http://www.who.int/trialsearch/Trial2.aspx?TrialID=CTRI/2018/07/014843* (2018) <https://www.cochranelibrary.com/central/doi/10.1002/central/CN-01906603/full>  2. NCT03892603. Does The Type of Exercise Influence Outcome in Rotator Cuff Related Shoulder Pain. *https://clinicaltrials.gov/show/NCT03892603* (2019) <https://www.cochranelibrary.com/central/doi/10.1002/central/CN-01911951/full>  3. Maenhout A, Mahieu, N, De Muynck, M, De Wilde, L, Cools A. Eccentric training in patients with rotator cuff tendinopathy: a randomized controlled trial. *Physiotherapy (united kingdom)* (2011) 97:eS738‐. doi: [10.1016/j.physio.2011.04.002](https://doi.org/10.1016/j.physio.2011.04.002)  4. NCT05318209. Effect of Closed-chain Shoulder Girdle Scapular Depression Exercise on Shoulder Impingement Syndrome. *https://clinicaltrials.gov/show/NCT05318209* (2022) doi: [10.1002/central/CN-02392290](https://doi.org/10.1002/central/CN-02392290)  5. NCT02837848. Effect of Muscle Coactivation Strengthening for Rotator Cuff Tendinopathy. *https://clinicaltrials.gov/show/NCT02837848* (2016) <https://www.cochranelibrary.com/central/doi/10.1002/central/CN-01506909/full>  6. NCT02926443. Effectiveness of Supervised Motor Control Exercises on Rotator Cuff Tendinopathies. *https://clinicaltrials.gov/show/NCT02926443* (2016) <https://www.cochranelibrary.com/central/doi/10.1002/central/CN-01521388/full>  7. NCT03494192. Effects of Different Rehabilitation Programme on Pain, Function and AHD in Patients With SPS And Comparison With Healthy Controls. *https://clinicaltrials.gov/show/NCT03494192* (2018) <https://www.cochranelibrary.com/central/doi/10.1002/central/CN-01586132/full>  8. NCT05331963. Effects of Functional Kinesio Taping on Subacromial Space in Athletes With Rotator Cuff Tendinopathy. *https://clinicaltrials.gov/show/NCT05331963* (2022) doi: [10.1002/central/CN-02392639](https://doi.org/10.1002/central/CN-02392639)  9. NCT03566849. Effects of the Kinetic Chain Approach for Scapular Dyskinesis. *https://clinicaltrials.gov/show/NCT03566849* (2018) <https://www.cochranelibrary.com/central/doi/10.1002/central/CN-01660589/full>  10. NCT02670174. Influence of Kinetic Chain Training on the Treatment Outcome of Overhead Athletes With Impingement. *https://clinicaltrials.gov/show/NCT02670174* (2015) <https://www.cochranelibrary.com/central/doi/10.1002/central/CN-01555469/full>  11. CTRI/2018/05/013892. Physiotherapy treatment of shoulder pain in sports persons. *http://www.who.int/trialsearch/Trial2.aspx?TrialID=CTRI/2018/05/013892* (2018) <https://www.cochranelibrary.com/central/doi/10.1002/central/CN-01895712/full>  12. NCT04468594. Rigid Taping Versus Scapular Stabilizing Exercises in Subacromial Impingement Syndrome. *https://clinicaltrials.gov/show/NCT04468594* (2020) <https://www.cochranelibrary.com/central/doi/10.1002/central/CN-02134380/full>  13. ChiCTR-IOR-17012521. Scapular Dyskinesis of Subacromial Impingement syndrome and development of appropriate rehabilitation techniques. *http://www.who.int/trialsearch/Trial2.aspx?TrialID=ChiCTR-IOR-17012521* (2017) <https://www.cochranelibrary.com/central/doi/10.1002/central/CN-01887239/full>  14. NCT04813757. Scapular Upward Rotation Focused Treatment for Patient With Shoulder Impingement Syndrome and a Positive Scapular Assistance Test. *https://clinicaltrials.gov/show/NCT04813757* (2021) doi: [10.1002/central/CN-02253153](https://doi.org/10.1002/central/CN-02253153)  15. RBR-4d5zcg. Specific versus general exercises program in patients with shoulder impingement syndrome. *http://www.who.int/trialsearch/Trial2.aspx?TrialID=RBR-4d5zcg* (2020) <https://www.cochranelibrary.com/central/doi/10.1002/central/CN-02173691/full>  16. CTRI/2021/03/032109. The Effect of Muscle Energy Technique on Shoulder Muscle Flexibility, Pain, Movement and Function in Patients with Subacromial Impingement Syndrome. *https://trialsearch.who.int/Trial2.aspx?TrialID=CTRI/2021/03/032109* (2021) doi: [10.1002/central/CN-02255552](https://doi.org/10.1002/central/CN-02255552)  17. NCT04273568. The Effect of Scapular Proprioceptive Neuromuscular Facilitation Techniques on Pain and Functionality in Subacromial Impingement Syndrome. *https://clinicaltrials.gov/show/NCT04273568* (2020) <https://www.cochranelibrary.com/central/doi/10.1002/central/CN-02088246/full>  18. ChiCTR2100044332. The effect of scapular stabilization exercise on the treatment of scapular dyskinesis in patients with periarthritis of shoulder. *http://www.who.int/trialsearch/Trial2.aspx?TrialID=ChiCTR2100044332* (2022) doi: [10.1002/central/CN-02438210](https://doi.org/10.1002/central/CN-02438210)  19. ChiCTR-IOR-17012630. The effect of scapular-focused programme for patients with rotator cuff tendinopathy and scapular dyskinesis. *http://www.who.int/trialsearch/Trial2.aspx?TrialID=ChiCTR-IOR-17012630* (2017) <https://www.cochranelibrary.com/central/doi/10.1002/central/CN-01887207/full>  20. Yuksel E, Yesilyaprak S. The effectiveness of scapular stabilization exercises in patients with subacromial impingement syndrome and scapular dyskinesis. *Annals of the rheumatic diseases* (2015) 74:1316. doi: [10.1136/annrheumdis-2015-eular.5594](https://doi.org/10.1136/annrheumdis-2015-eular.5594)  21. NCT04493190. The Effects of Short-term Scapular Control Training in Overhead Athletes With Shoulder Impingement Syndrome. *https://clinicaltrials.gov/show/NCT04493190* (2020) <https://www.cochranelibrary.com/central/doi/10.1002/central/CN-02145716/full> |
| --- | --- |
| Non-English | 1. Ferreiro Marzoa I, Veiga Suárez M, Guerra Peña JL, Rey Veiga S, Paz Esquete J, Tobío Iglesias A. Tratamiento rehabilitador del hombro doloroso. *Rehabilitación* (2005) 39:113–120. doi: [10.1016/S0048-7120(05)74328-0](https://doi.org/10.1016/S0048-7120(05)74328-0) |
| **Not fitting eligibility criteria:** | |
| Population | 1. McNeely ML, Parliament, M, Courneya, KS, Seikaly, H, Jha, N, Scrimger, R, Hanson J. A pilot study of a randomized controlled trial to evaluate the effects of progressive resistance exercise training on shoulder dysfunction caused by spinal accessory neurapraxia/neurectomy in head and neck cancer survivors. *Head & neck* (2004) 26:518‐530. doi: [10.1002/hed.20010](https://doi.org/10.1002/hed.20010)  2. McNeely ML, Parliament, M, Courneya, KS, Seikaly, H, Jha, N, Scrimger, R, Hanson J. A pilot study of a randomized controlled trial to evaluate the effects of progressive resistance exercise training on shoulder dysfunction caused by spinal accessory neurapraxia/neurectomy in head and neck cancer survivors. *Head & neck* (2004) 26:518‐530. doi: [10.1002/hed.20010](https://doi.org/10.1002/hed.20010)  3. Buttagat V, Taepa N, Suwannived N, Rattanachan N. Effects of scapular stabilization exercise on pain related parameters in patients with scapulocostal syndrome: A randomized controlled trial. *Journal of bodywork and movement therapies* (2016) 20:115–122. doi: [10.1016/j.jbmt.2015.07.036](https://doi.org/10.1016/j.jbmt.2015.07.036)  4. McGarvey AC, Hoffman GR, Osmotherly PG, Chiarelli PE. Maximizing shoulder function after accessory nerve injury and neck dissection surgery: A multicenter randomized controlled trial. *Head Neck* (2015) 37:1022–1031. doi: [10.1002/hed.23712](https://doi.org/10.1002/hed.23712)  5. Chen YH, Lin, CR, Liang, WA, Huang C. Motor control integrated into muscle strengthening exercises has more effects on scapular muscle activities and joint range of motion before initiation of radiotherapy in oral cancer survivors with neck dissection: a randomized controlled trial. *PloS one* (2020) 15:e0237133. doi: [10.1371/journal.pone.0237133](https://doi.org/10.1371/journal.pone.0237133) |
| Intervention | 1. ACTRN12616001676404. A pilot randomised controlled trial comparing three different physiotherapy interventions to treat rotator cuff tendinopathy/subacromial pain syndrome.*http://www.who.int/trialsearch/Trial2.aspx?TrialID=ACTRN12616001676404* (2016) <https://www.cochranelibrary.com/central/doi/10.1002/central/CN-01805540/full>  2. Antunes A, Neto, S, Carnide, F, Matias R. Biofeedback-assisted learning of scapula dynamic control is influenced by the dimensionality of the feedback information. *Gait & posture* (2016) 49:274‐. doi: [10.1016/j.gaitpost.2016.07.326](https://doi.org/10.1016/j.gaitpost.2016.07.326)  3. Senbursa G, Baltaci, G, Atay A. Comparison of conservative treatment with and without manual physical therapy for patients with shoulder impingement syndrome: a prospective, randomized clinical trial. *Knee surgery, sports traumatology, arthroscopy* (2007) 15:915‐921. doi: [10.1007/s00167-007-0288-x](https://doi.org/10.1007/s00167-007-0288-x)  4. Pekyavas NO, Ergun N. Comparison of virtual reality exergaming and home exercise programs in patients with subacromial impingement syndrome and scapular dyskinesis: short term effect. *Acta orthopaedica et traumatologica turcica* (2017) 51:238‐242. doi: [10.1016/j.aott.2017.03.008](https://doi.org/10.1016/j.aott.2017.03.008)  5. Stevenson K, Jackson, S, Shufflebotham, J, Roddy, E, Foster N. Development and delivery of a physiotherapist-led exercise intervention in a randomised controlled trial for subacromial impingement syndrome (the SUPPORT trial). *Physiotherapy* (2017) 103:379‐386. doi: [10.1016/j.physio.2017.03.005](https://doi.org/10.1016/j.physio.2017.03.005)  6. Jeon N-Y, Chon S-C. Effect of glenohumeral stabilization exercises combined with scapular stabilization on shoulder function in patients with shoulder pain: A randomized controlled experimenter-blinded study. *Journal of Back and Musculoskeletal Rehabilitation* (2018) 31:259–265. doi: [10.3233/BMR-169612](https://doi.org/10.3233/BMR-169612)  7. Holmgren T, Hallgren HB, Öberg B, Adolfsson L, Johansson K. Effect of specific exercise strategy on need for surgery in patients with subacromial impingement syndrome: randomised controlled study. *BMJ* (2012) 344:e787. doi: [10.1136/bmj.e787](https://doi.org/10.1136/bmj.e787)  8. Clausen MB, Hölmich P, Rathleff M, Bandholm T, Christensen KB, Zebis MK, Thorborg K. Effectiveness of Adding a Large Dose of Shoulder Strengthening to Current Nonoperative Care for Subacromial Impingement: A Pragmatic, Double-Blind Randomized Controlled Trial (SExSI Trial). *Am J Sports Med* (2021) 49:3040–3049. doi: [10.1177/03635465211016008](https://doi.org/10.1177/03635465211016008)  9. Dabholkar SA, Yardi SS. Effects of scapular muscle strengthening on shoulder function and disability in shoulder impingement syndrome (SIS) -A Randomized controlled trial. *International Journal of Therapies & Rehabilitation Research* (2015) 4:26–30.  10. Bennell K, Coburn S, Wee E, Green S, Harris A, Forbes A, Buchbinder R. Efficacy and cost-effectiveness of a physiotherapy program for chronic rotator cuff pathology: A protocol for a randomised, double-blind, placebo-controlled trial. *Bmc Musculoskeletal Disorders* (2007) 8:86. doi: [10.1186/1471-2474-8-86](https://doi.org/10.1186/1471-2474-8-86)  11. Huang HY, Lin, JJ, Guo, YL, Wang, WT, Chen Y. EMG biofeedback effectiveness to alter muscle activity pattern and scapular kinematics in subjects with and without shoulder impingement. *Journal of electromyography and kinesiology* (2013) 23:267‐274. doi: [10.1016/j.jelekin.2012.09.007](https://doi.org/10.1016/j.jelekin.2012.09.007)  12. Huang T-S, Du, W-Y, Wang, T-G, Tsai, Y-S, Yang, J-L, Huang, CY, Lin J-J. Progressive conscious control of scapular orientation with video feedback has improvement in muscle balance ratio in patients with scapular dyskinesis: a randomized controlled trial. *Journal of shoulder and elbow surgery* (2018) (no pagination): doi: [10.1016/j.jse.2018.04.006](https://doi.org/10.1016/j.jse.2018.04.006)  13. Gutiérrez-Espinoza H, Araya-Quintanilla, F, Zavala-González, J, Gana-Hervias, G, Martínez-Vizcaino, V, Álvarez-Bueno, C, Cavero-Redondo I. Rationale and methods of a randomized clinical trial to compare specific exercise programs versus home exercises in patients with subacromial impingement syndrome. *Medicine* (2019) 98:e16139. doi: [10.1097/MD.0000000000016139](https://doi.org/10.1097/MD.0000000000016139)  14. Hotta GH, Santos AL, McQuade KJ, de Oliveira AS. Scapular-focused exercise treatment protocol for shoulder impingement symptoms: Three-dimensional scapular kinematics analysis. *Clinical Biomechanics* (2018) 51:76–81. doi: [10.1016/j.clinbiomech.2017.12.005](https://doi.org/10.1016/j.clinbiomech.2017.12.005)  15. Du WY, Huang, TS, Chiu, YC, Mao, SJ, Hung, LW, Liu, MF, Yang, JL, Lin J. Single-Session Video and Electromyography Feedback in Overhead Athletes With Scapular Dyskinesis and Impingement Syndrome. *Journal of athletic training* (2020) 55:265‐273. doi: [10.4085/1062-6050-490-18](https://doi.org/10.4085/1062-6050-490-18)  16. NCT02747251. Strengthening Exercises in Shoulder Impingement (SExSI) Trial. *https://clinicaltrials.gov/show/NCT02747251* (2016) <https://www.cochranelibrary.com/central/doi/10.1002/central/CN-01557584/full>  17. Aytar A, Baltaci G, Uhl T, Tuzun H, Oztop P, Karatas M. The Effects of Scapular Mobilization in Patients With Subacromial Impingement Syndrome: A Randomized, Double-Blind, Placebo-Controlled Clinical Trial. *Journal of Sport Rehabilitation* (2015) 24:116–129. doi: [10.1123/jsr.2013-0120](https://doi.org/10.1123/jsr.2013-0120)  18. Holmgren T, Oberg, B, Bjornsson, H, Adolfsson, L, Johansson K. The efficacy of standardized exercises for the rotator cuff and scapular stabilizers in patients with subacromial impingement syndrome-RCT study. *Physiotherapy (united kingdom)* (2011) 97:eS497. doi: [10.1016/j.physio.2011.04.002](https://doi.org/10.1016/j.physio.2011.04.002) |
| Study Type | 1. Dewhurst A. An exploration of evidence-based exercises for shoulder impingement syndrome. *International Musculoskeletal Medicine* (2010) 32:111–116.  2. Shankar P, Jayaprakasan P, Devi R. EFFECT OF SCAPULAR STABILISATION EXERCISES FOR TYPE 2 SCAPULAR DYSKINESIS IN SUBJECTS WITH SHOULDER IMPINGEMENT. *International Journal of Physiotherapy* (2016)106–110. doi: [10.15621/ijphy/2016/v3i1/88921](https://doi.org/10.15621/ijphy/2016/v3i1/88921)  3. Berg OK, Paulsberg F, Brabant C, Arabsolghar K, Ronglan S, Bjørnsen N, Tørhaug T, Granviken F, Gismervik S, Hoff J. High-Intensity Shoulder Abduction Exercise in Subacromial Pain Syndrome. *Medicine & Science in Sports & Exercise* (2021) 53:1. doi: [10.1249/MSS.0000000000002436](https://doi.org/10.1249/MSS.0000000000002436)  4. Vallés-Carrascosa E, Gallego-Izquierdo T, Jiménez-Rejano JJ, Plaza-Manzano G, Pecos-Martín D, Hita-Contreras F, Achalandabaso Ochoa A. Pain, motion and function comparison of two exercise protocols for the rotator cuff and scapular stabilizers in patients with subacromial syndrome. *Journal of Hand Therapy* (2018) 31:227–237. doi: [10.1016/j.jht.2017.11.041](https://doi.org/10.1016/j.jht.2017.11.041)  5. San Juan JG, Gunderson SR, Kane-Ronning K, Suprak DN. Scapular kinematic is altered after electromyography biofeedback training. *Journal of Biomechanics* (2016) 49:1881–1886.  6. Björnsson Hallgren HC, Adolfsson LE, Johansson K, Öberg B, Peterson A, Holmgren TM. Specific exercises for subacromial pain. *Acta Orthop* (2017) 88:600–605. doi: [10.1080/17453674.2017.1364069](https://doi.org/10.1080/17453674.2017.1364069) |

**
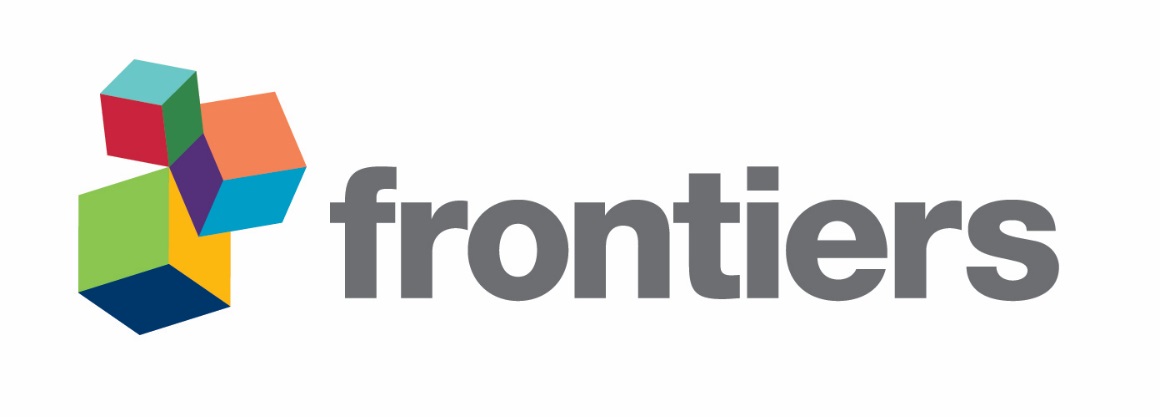
**
